# Economic costs for outpatient treatment of eating disorders in Japan

**DOI:** 10.1186/s40337-023-00864-2

**Published:** 2023-08-14

**Authors:** Ken Kurisu, Nobuhiro Nohara, Shuji Inada, Makoto Otani, Haruko Noguchi, Yuka Endo, Yasuhiro Sato, Shin Fukudo, Michiko Nakazato, Tsuneo Yamauchi, Tomoko Harada, Koki Inoue, Tomokazu Hata, Shu Takakura, Nobuyuki Sudo, Naoko Iida, Yuki Mizuhara, Yoshihisa Wada, Tetsuya Ando, Kazuhiro Yoshiuchi

**Affiliations:** 1 Department of Stress Sciences and Psychosomatic Medicine, Graduate School of Medicine, The University of Tokyo, Tokyo, Japan; 2 Department of Psychosomatic Medicine, Kindai University Hospital, Osaka, Japan; 3 Department of Psychosomatic Medicine, The University of Tokyo Hospital, Tokyo, Japan; 4 Faculty of Political Science and Economics, Waseda University, Tokyo, Japan; 5 Department of Psychosomatic Medicine, Tohoku University Hospital, Sendai, Miyagi Japan; 6 Department of Psychosomatic Medicine, Tohoku University Graduate School of Medicine, Sendai, Miyagi Japan; 7 Department of Psychiatry, International University of Health and Welfare, Chiba, Japan; 8 Department of Psychiatry, Chiba University Graduate School of Medicine, Chiba, Japan; 9 Department of Neuropsychiatry, Osaka Metropolitan University Hospital, Osaka, Japan; 10 Department of Psychosomatic Medicine, Kyushu University Hospital, Fukuoka, Japan; 11 Department of Psychosomatic Medicine, Graduate School of Medical Sciences, Kyushu University, Fukuoka, Japan; 12 Department of Psychiatry, Graduate School of Medical Science, Kyoto Prefectural University of Medicine, Kyoto, Japan; 13 Kyoto Prefectural Support Center of Child Development, Kyoto, Japan; 14 Fuchu Mikumari Hospital, Hiroshima, Japan; 15 Department of Psychosomatic Medicine, Narita Hospital, International University of Health and Welfare, Chiba, Japan

**Keywords:** Economic costs, Reimbursement, Eating disorders, Psychosomatic Medicine, Eating Disorder Examination Questionnaire, Outpatients

## Abstract

**Background:**

Few studies have examined the economic costs of outpatient care for eating disorders in Japan. This study aimed to clarify the reimbursement for outpatient treatment of eating disorders and compare the costs between the departments of Psychosomatic Medicine and Psychiatry in Japan.

**Method:**

A multicenter, prospective, observational study of patients with an eating disorder was conducted in the Psychosomatic Medicine departments of three centers and the Psychiatry departments of another three centers in Japan. We analyzed medical reimbursement for an outpatient revisit, time of clinical interviews, and the treatment outcome measured by the Eating Disorder Examination Questionnaire (EDE-Q) global scores and body mass index (BMI) at 3 months. Multivariate linear regression models were performed to adjust for covariates.

**Results:**

This study included 188 patients in the Psychosomatic Medicine departments and 68 in the Psychiatry departments. The average reimbursement cost for an outpatient revisit was 4670 yen. Even after controlling for covariates, the Psychosomatic Medicine departments had lower reimbursement points per minute of interviews than the Psychiatry departments (coefficient = − 23.86; 95% confidence interval = − 32.09 to − 15.63; *P* < 0.001). In contrast, EDE-Q global scores and BMI at 3 months were not significantly different between these departments.

**Conclusions:**

This study clarifies the economic costs of treating outpatients with eating disorders in Japan. The medical reimbursement points per interview minute were lower in Psychosomatic Medicine departments than in Psychiatry departments, while there were no apparent differences in the treatment outcomes. Addressing this issue is necessary to provide an adequate healthcare system for patients with eating disorders in Japan.

## Abstract (plain language summary)

This study examined the cost of outpatient care for eating disorders in Japan, comparing treatment costs between the Psychosomatic Medicine and Psychiatry departments. The actual cost of outpatient care for eating disorders in Japan was clarified. The results indicate that Psychosomatic Medicine departments have lower reimbursement points per interview time compared to the Psychiatry departments, but there were no noticeable differences in treatment outcomes between the two. This highlights the need to address this cost difference to improve the healthcare system for patients with eating disorders in Japan.

## Background

Eating disorders are highly prevalent, especially among young women [1], and the incidence of eating disorders has reportedly increased since the COVID-19 pandemic [2]. Mortality rates have been reported to be high in patients with anorexia nervosa, and binge-eating or purging behaviors can severely impair quality of life, resulting in substantial social and economic costs [3]. While cognitive behavior therapy is an evidence-based treatment for eating disorders [4], it requires a substantial amount of time [5].

A systematic review has summarized the medical expenses of patients with eating disorders in the United States, the United Kingdom, Germany, Finland, and Canada [6]. This review has reported that the annual medical costs for anorexia nervosa, bulimia nervosa, and binge-eating disorder range from 2993 to 55,270, 888 to 18,823, and 1762 to 2902 euros (at the 2014 exchange rate), respectively. This review also found that the annual cost of outpatient care for eating disorders varied from 3669 to 27,889 euros (at the 2014 exchange rate) in those countries [6–9]. However, few studies from Japan have investigated such medical costs, as well as the time required for clinical interviews, for patients with eating disorders. This scarcity of reports on economic costs may result from the limited availability of facilities that provide treatment for eating disorders throughout Japan [10].

The Psychosomatic Medicine department, a part of the Internal Medicine department in Japan, focuses primarily on physical diseases that require behavioral medicine intervention, such as obesity, type 2 diabetes, and irritable bowel syndrome [11]. In Japan, psychosomatic physicians, as well as psychiatrists, play a leading role in the treatment and research of eating disorders, exemplified by conducting clinical trials of cognitive behavior therapy [12]. Psychosomatic physicians typically provide psychosomatic therapy (1100 yen for new patients; 800 yen for follow-up visits), while psychiatrists usually provide outpatient psychotherapy (4000 yen for ≥ 30 min interviews; 3300 yen for < 30 min) [13], with a fourfold difference in reimbursement between them.

In this study, we analyzed the medical reimbursement in centers that provide treatment for eating disorders in Japan, aiming to clarify the actual reimbursement for outpatient treatment of eating disorders in Japan and compare those between the departments of Psychosomatic Medicine and Psychiatry.

## Methods

### Study design and participants

This was a multicenter, prospective, observational study that included patients with an eating disorder diagnosed with the Diagnostic and Statistical Manual of Mental Disorders, 5th Edition [14] who were receiving treatment at the Psychosomatic Medicine department of three centers (the University of Tokyo Hospital, Kyushu University Hospital, and Tohoku University Hospital) and at the Psychiatry department of another three centers (Osaka Metropolitan University Hospital, Kyoto Prefectural University of Medicine Hospital, and Chiba University Hospital). Most of the staff in the Psychosomatic Medicine departments were specialists in internal medicine. Medical care in all facilities was provided by members of the Japan Society for Eating Disorders or under their supervision, and treatment was largely in accordance with the standard treatment guidelines for eating disorders [4]. Data were collected from October 2015 to March 2017.

Clinical data, including age, sex, disease type, duration of diseases, height, and weight, were collected from routine practices, and the amount of time for clinical interviews and the reimbursement per visit were also obtained. The reimbursement points, which were obtained from the medical affairs departments of each facility, included the doctors’ consultation fees, costs for laboratory, physiological, and radiology tests, prescription fees (usually not including the cost of the drugs themselves), and nutrition counseling charges. At the start of the study and 3 months after, body weight and the Japanese version of the Eating Disorder Examination Questionnaire (EDE-Q) 6.0 [15] were measured as indicators of treatment response.

All participants provided written consent to participate in the study. The protocol was approved by the Institutional Review Board of the University of Tokyo (Approval Number: 10779-(4)).

### Statistical analyses

Medical reimbursement per visit and minutes for clinical interviews were analyzed using data from outpatient follow-up visits. Changes in body mass index (BMI) were analyzed for patients who were underweight (i.e., BMI < 18.5) at the beginning of the study.

To compare variables between the departments of Psychosomatic Medicine and Psychiatry, we applied a t-test (Student or Welch) or the Mann–Whitney U test for continuous variables, such as BMI, after examining variance homogeneity using the F test and normality using the Kolmogorov–Smirnov test. This comparison for continuous variables excluded patients with missing values. We also used the Chi-squared test or Fisher’s exact test to compare the proportions of categorical variables, such as the type of diseases, between the departments.

We examined the changes in EDE-Q global score and BMI between pre-treatment and post-treatment in the overall participants. This assessment of BMI was conducted only among underweight patients. The Kolmogorov–Smirnov test was used to assess normality, followed by either the Wilcoxon signed-rank test or a paired *t* test, as appropriate.

Multivariate linear regression models were developed to analyze medical reimbursement per amount of time for clinical interviews, the EDE-Q global score at 3 months, and BMI at 3 months as the outcomes. The models always used a binary variable representing the Psychosomatic Medicine or Psychiatry department. Other variables were determined to be included in the model by checking improvements in the Akaike Information Criterion and the number of participants excluded due to missing values.

All statistical analyses were performed using R version 4.2.2 (R Foundation for Statistical Computing, Vienna, Austria, 2022). Statistical significance was set at *P* < 0.05.

## Results

### Descriptive statistics

Data on 188 patients in the Psychosomatic Medicine department and 68 in the Psychiatry department were collected. The ages ranged from 11 to 55 years. The average reimbursement point for an outpatient revisit was 467 points (standard deviation: 390 points), equivalent to 4670 yen. Given the 30% individual charge for patients aged between 6 and 69 years in Japan [16], the average expense for patients themselves was approximately 1400 yen. Additionally, the average number of monthly visits was 1.65 days (standard deviation: 0.91 days). Table 1 shows the mean reimbursement points separated by disease type.

**Table 1:** Medical reimbursement points for an outpatient revisit of patients with eating disorders

	N	Reimbursement points per single revisit^a^ Mean (SD)	Number of monthly visits Mean (SD)
Total	152	467.25 (390.22)	1.65 (0.91)
ANR	45	523.39 (475.27)	1.56 (0.84)
ANBP	48	487.97 (391.92)	1.77 (0.97)
BN	42	366.14 (210.93)	1.79 (1.00)
BED	3	196.33 (180.09)	1.33 (0.58)
Other/Missing	14	577.21 (477.55)	1.21 (0.43)

*SD* standard deviation, *ANR* anorexia nervosa restricting type, *ANBP* anorexia nervosa binge-purging type, *BN* bulimia nervosa, *BED* binge eating disorders

^a^One point of medical reimbursement is equivalent to ¥10

Table 2 describes the comparative results in patients without missing values in continuous variables. The departments of Psychosomatic Medicine had lower reimbursement for outpatient revisits (mean, 462.55 vs. 476.56 points; *P* < 0.001), longer duration of clinical interviews (mean, 28.05 vs. 17.26 min; *P* < 0.001), and lower reimbursement per minute of interview (mean, 18.12 vs. 31.95 points per minute; *P* < 0.001) than the Psychiatry departments. In contrast, no significant differences were observed in the 3-month changes in the EDE-Q global score (mean, − 0.27 vs. − 0.33; *P* = 0.73) and in the BMI changes among underweight patients (mean, 0.06 vs. − 0.01 kg/m^2^; *P* = 0.76) between the departments.

**Table 2:** Comparative analysis between the Psychosomatic Medicine and Psychiatry departments

	Psychosomatic medicine	Psychiatry	*P* value
Age (years)	(N = 163)	(N = 65)	
Mean (SD)	29.59 (10.79)	29.22 (9.50)	0.81^a^
Median (Range)	27 (11–55)	27 (13–47)	
Sex, N (%)	(N = 188)	(N = 68)	
Female	162 (86.2)	61 (89.7)	0.037^b^
Male	3 (1.6)	4 (5.9)	
Missing	23 (12.2)	3 (4.4)	
Disease type, N (%)	(N = 188)	(N = 68)	
ANR	60 (31.9)	22 (32.4)	0.14^b^
ANBP	51 (27.1)	21 (30.9)	
BN	36 (19.1)	16 (23.5)	
BED	1 (0.5)	2 (2.9)	
Other/missing	40 (21.3)	7 (10.3)	
Disease duration (months)	(N = 147)	(N = 65)	
Mean (SD)	123.95 (105.97)	107.29 (92.00)	0.35^c^
Median (Range)	92 (0–432)	84 (11–353)	
Medical reimbursement points for an outpatient revisit	(N = 101)	(N = 51)	
Mean (SD)	462.55 (463.87)	476.56 (171.40)	< 0.001^c^
Median (Range)	223 (113–2214)	471 (73–1021)	
Clinical interview time (min)	(N = 102)	(N = 31)	
Mean (SD)	28.05 (8.82)	17.26 (10.07)	< 0.001^c^
Median (range)	30 (10–60)	15 (5–45)	
Reimbursement points per interview minute	(N = 101)	(N = 31)	
Mean (SD)	18.12 (18.75)	31.95 (11.57)	< 0.001^c^
Median (range)	10.43 (2.55–95.53)	31.40 (11.28–61.20)	
EDE-Q global score at baseline	(N = 126)	(N = 47)	
Mean (SD)	2.41 (1.44)	2.98 (1.37)	0.020^a^
Median (range)	2.40 (0.00–5.21)	3.36 (0.22–5.09)	
EDE-Q global score at 3 months	(N = 65)	(N = 25)	
Mean (SD)	1.93 (1.34)	2.43 (1.43)	0.12^a^
Median (range)	1.85 (0.00–4.95)	2.52 (0.06–5.01)	
Changes in EDE-Q global score	(N = 59)	(N = 23)	
Mean (SD)	− 0.27 (0.70)	− 0.33 (0.89)	0.73^a^
Median (range)	− 0.12 (− 1.91 to 1.15)	− 0.02 (− 3.19 to 0.56)	
BMI at baseline	(N = 90)	(N = 38)	
Mean (SD)	15.01 (1.90)	15.05 (2.36)	0.92^a^
Median (range)	15.07 (10.54–18.38)	14.88 (9.21–18.44)	
BMI at 3 months	(N = 43)	(N = 19)	
Mean (SD)	14.93 (1.83)	14.20 (1.89)	0.16^a^
Median (range)	14.86 (10.62–18.67)	14.38 (9.33–17.33)	
Changes in BMI	(N = 43)	(N = 19)	
Mean (SD)	0.06 (0.74)	− 0.01 (0.75)	0.76^a^
Median (range)	0.00 (− 2.20 to 2.04)	0.00 (− 2.60 to 0.86)	

Each cell includes the number of comparative samples within parentheses. Medical reimbursement points are presented for a single outpatient revisit. Results for BMI are presented for patients who were underweight (BMI < 18.5) at the beginning of the study

*SD* standard deviation, *BMI* body mass index, *EDE-Q* eating disorder examination questionnaire 6.0, *ANR* anorexia nervosa restricting type, *ANBP* anorexia nervosa binge-purging type, *BN* bulimia nervosa, *BED* binge eating disorders

^a^Student *t* test

^b^Fisher’s exact test

^c^Mann–Whitney U test

The EDE-Q global scores showed a significant improvement over a 3-month period in the overall participants (mean difference = − 0.287; 95% confidence interval [CI] = − 0.453 to − 0.123; *P* < 0.001 using paired t-test). The changes in BMI were small and not statistically significant (mean difference = 0.036; *P* = 0.20 using the Wilcoxon signed-rank test).

### Multivariate regression models

The multivariate regression analysis for the reimbursement per minute finally incorporated departments and baseline EDE-Q global scores as explanatory variables (N = 95). It indicated that the reimbursement per minute was significantly lower in the departments of Psychosomatic Medicine (coefficient = − 23.86; 95% CI = − 32.09 to − 15.63; *P* < 0.001) and also inversely correlated with baseline EDE-Q global scores (coefficient = − 4.21; 95% CI = − 6.58 to − 1.84; *P* < 0.001).

In the multivariate linear regression model for the EDE-Q global score at 3 months, departments and baseline EDE-Q global scores were included as explanatory variables (N = 82). It revealed that the scores at 3 months in the Psychosomatic Medicine departments were not significantly different from those in the Psychiatry departments (coefficient = − 0.05; 95% CI = − 0.41 to 0.30; *P* = 0.76) and that baseline EDE-Q global scores were positively associated with the outcome (coefficient = 0.82; 95% CI = 0.71–0.93; *P* < 0.001).

The multivariate linear regression analysis for BMI at 3 months among underweight patients included departments, age, and the baseline BMI as explanatory variables (N = 62). BMI at 3 months in the Psychosomatic Medicine departments was not significantly different from that in the Psychiatry departments (coefficient = 0.09; 95% CI = − 0.28 to 0.46; *P* = 0.63). Additionally, patients of younger age (coefficient = − 0.02; 95% CI = − 0.04 to − 0.01; *P* = 0.011) and those with higher baseline BMI (coefficient = 0.84; 95% CI = 0.75–0.93; *P* < 0.001) tended to have higher BMI at 3 months.

## Discussion

This study revealed the economic cost of treating outpatients with eating disorders in Japan. We found that the reimbursement points per interview minute in the Psychosomatic Medicine departments were significantly lower than that in the Psychiatry departments, in contrast to no significant differences in EDE-Q global scores and BMI at 3 months in patients between the two departments.

The average reimbursement for a single outpatient revisit was 4670 yen, and the average number of monthly visits was 1.65 days. Therefore, when assuming continuous visits throughout the year, the annual outpatient cost is approximately 92,500 yen. This is equivalent to 754 euros or 794 US dollars based on the January 1, 2017, exchange rate. Studies from other countries have reported annual outpatient costs ranging from 3669 to 27,889 euros [6–9], which are equivalent to 4337–32,969 euros or 4569–34,727 US dollars based on the January 1, 2017, exchange rate. Even considering the fact that we excluded the initial outpatient cost from the analysis, the medical reimbursement for treating eating disorders in Japan seems much lower than that in other countries. However, careful interpretation is required for this comparison, as the composition of costs and the healthcare systems can vary considerably across countries.

A significant difference in the reimbursement for treating eating disorders between the departments was observed without apparent differences in treatment outcomes. Given the limited availability of facilities that can treat eating disorders in Japan [10] and the presumably increased prevalence of patients [2], the imbalance between departments should be addressed to establish a consistent and adequate healthcare system for patients with eating disorders nationwide. A plausible strategy is to implement dedicated treatment scores for outpatients with eating disorders, similar to those for inpatient care [13].

While EDE-Q global scores tended to improve at 3 months among the overall participants, we found no significant differences in the scores or BMI at 3 months between the two departments. However, treatment of eating disorders typically extends over 20 weeks, and many studies follow participants for approximately 1 year [17, 18]. Thus, further research is warranted to determine the effectiveness of treatment at each institution and differences in treatment outcomes among departments.

The multivariate regression model for the medical reimbursement points per minute showed that higher baseline EDE-Q scores were associated with lower point-minute ratios. This suggests that patients with more severe psychopathology of eating disorders may require more time for medical interviews, which were not factored into the medical costs, such as time for psychoeducation. In addition to setting the medical reimbursement specific to eating disorders, differentiating the points according to the severity of the disease is necessary.

We also found that baseline EDE-Q global scores and BMI were associated with those values at 3 months, respectively. A previous study that developed machine learning models predicting treatment outcomes for eating disorders demonstrated that these baseline values are among the most important variables for prediction [19], which is consistent with our findings. Additionally, younger patients showed more weight gain, though the effect size was small. This result is consistent with previous research indicating that patients with anorexia nervosa with a longer disease duration, which is usually correlated with older age, have a poorer prognosis [20].

This study had several limitations. First, the dataset had numerous missing values, and discrepancies existed between the number of overall participants (188 in Psychosomatic Medicine and 68 in Psychiatry) and comparable participants (e.g., 101 in Psychosomatic Medicine and 51 in Psychiatry for comparison of reimbursement points), which could have influenced the results. Second, treatment outcomes were only examined at 3 months; thus, analyses with a longer follow-up would be desirable. Third, the study participants were only from medical institutions that are relatively experienced in treating eating disorders. The results from medical clinics and facilities that treat small numbers of patients and have less experience remain unknown. Fourth, we analyzed the medical reimbursements at each participating institution in this study; however, we were unable to consider any costs associated with other facilities that the patients might have visited. Finally, the comparison between Psychosomatic Medicine and Psychiatry departments in this study was not based on randomized assignment, necessitating careful interpretation of the result.

## Conclusions

In this study, we clarify the costs of treating outpatients with eating disorders in Japan. The medical reimbursement points per interview minute were lower in the departments of Psychosomatic Medicine than those in Psychiatry, regardless of no significant differences in treatment outcomes. Addressing this issue through implementing specified medical reimbursement points for eating disorders in outpatient settings is necessary to provide an adequate healthcare system for patients with eating disorders in Japan.

## Data Availability

The dataset is available from the corresponding author upon reasonable request.

## Abbreviations

BMI: Body mass index
EDE-Q: Eating Disorder Examination Questionnaire
